# Fetal Tracheal Occlusion Increases Lung Basal Cells *via* Increased Yap Signaling

**DOI:** 10.3389/fped.2021.780166

**Published:** 2022-02-22

**Authors:** Vincent Serapiglia, Chad A. Stephens, Rashika Joshi, Emrah Aydin, Marc Oria, Mario Marotta, Jose L. Peiro, Brian M. Varisco

**Affiliations:** ^1^School of Medicine, Northeast Ohio College of Medicine, Northeast Ohio Medical University, Rootstown Township, OH, United States; ^2^Department of Psychiatry, University of Illinois at Chicago, Chicago, IL, United States; ^3^Division of Critical Care Medicine, Department of Pediatrics, Cincinnati Children's Hospital Medical Center, Cincinnati, OH, United States; ^4^Department of Pediatric Surgery, Tekirdag Namik Kemal University School of Medicine, Tekirdag, Turkey; ^5^Center for Fetal and Placental Research, Cincinnati Children's Hospital Medical Center (CCHMC), Cincinnati, OH, United States; ^6^Department of Pediatrics, College of Medicine, University of Cincinnati, Cincinnati, OH, United States; ^7^Bioengineering, Cell Therapy and Surgery in Congenital Malformations Laboratory, Vall d'Hebron Hospital Research Institute, Universitat Autònoma de Barcelona, Barcelona, Spain

**Keywords:** fetal tracheal occlusion, congenital diaphragm hernia, lung development, basal cell, Yap (Hippo) signaling, mechanotransduction

## Abstract

Fetal endoscopic tracheal occlusion (FETO) is an emerging surgical therapy for congenital diaphragmatic hernia (CDH). Ovine and rabbit data suggested altered lung epithelial cell populations after tracheal occlusion (TO) with transcriptomic signatures implicating basal cells. To test this hypothesis, we deconvolved mRNA sequencing (mRNA-seq) data and used quantitative image analysis in fetal rabbit lung TO, which had increased basal cells and reduced ciliated cells after TO. In a fetal mouse TO model, flow cytometry showed increased basal cells, and immunohistochemistry demonstrated basal cell extension to subpleural airways. Nuclear Yap, a known regulator of basal cell fate, was increased in TO lung, and Yap ablation on the lung epithelium abrogated TO-mediated basal cell expansion. mRNA-seq of TO lung showed increased activity of downstream Yap genes. Human lung specimens with congenital and fetal tracheal occlusion had clusters of subpleural basal cells that were not present in the control. TO increases lung epithelial cell nuclear Yap, leading to basal cell expansion.

## Introduction

Congenital diaphragmatic hernia (CDH) results from the failed fusion of the pleuroperitoneal folds in the 6th to the 8th week of gestation in humans and occurs in 1:2,000 births (1). In CDH, abdominal contents herniate into the thorax, causing ipsilateral and contralateral lung hypoplasia and pulmonary vascular pruning. The morbidity and mortality associated with CDH are principally determined by the size of the defect and the laterality, with right-sided hernia experiencing worse outcomes. The most severe defects account for 20–35% of all CDH and have 50% mortality with severe respiratory morbidity in survivors (2). For this reason, a fetal surgical technique has been developed to improve fetal lung growth in CDH.

Fetal endoscopic tracheal occlusion (FETO) was first described in animal models of CDH by Harrison et al. (3), and human clinical trials have been ongoing for 35 years (1). Typically, FETO involves the placement of an endoluminal tracheal balloon at 26–29 weeks' gestation with fetoscopic removal at 34 weeks. Tracheal occlusion (TO) prevents the egress of epithelial secretions and causes lung expansion and growth. A meta-analysis of multiple small randomized trials indicated a survival benefit of FETO, but suggested continued respiratory morbidity (1). Larger randomized trials are ongoing. There are mixed reports of the impact of CDH on alveolar type II cell function (4–6) and a report that CDH reduces and TO increases TGF-β signaling and lung elastin synthesis (7). Two whole-lung gene expression profiling studies using a rabbit model reported complementary findings of decreased cell proliferation in CDH with restoration or excessive restoration of cell division with FETO (8, 9). Our group reported preferential proliferation in the epithelial compartment in FETO that was potentially EGFR-related (9). Proteomic analysis of tracheal fluid in an ovine model of CDH and FETO found that PI3K and mTOR signaling was increased with reduced abundance of ciliated cells in FETO (10). Adding to the complexity of animal models is a recent finding that, in a rabbit model of CDH and FETO, there are significant metabolic differences between and within lung lobes (11). A barrier to understanding the mechanistic underpinning of changes in CDH and TO has been the lack of a mouse TO model with high fetal survival rates. This obstacle has been recently overcome with the development of a transuterine mouse TO model (12, 13).

The Yes-associated protein (Yap) signaling cascade was first descried in *Drosophila* as the Hippo signaling pathway and is a key mechanosensitive integrator of several developmental processes in the lung epithelium. Yap is phosphorylated by large tumor suppressor kinases 1 and 2 (Lats1 and Lats2), which are phosphorylated by mammalian Ste20-like serine/threonine kinases 1 and 2 (Mst1 and Mst2), which are in turn phosphorylated by cytoskeleton-coupled proteins at epithelial cell apical membranes. Phosphorylated Yap is transcriptionally inactive and sequestered in the cytoplasm. Non-phosphorylated Yap is translocated into the nucleus, where it binds with TEAD (transcriptional enhancer factor domain proteins) factors, which are the enhancers of *Yap* target genes. The temporal and spatial roles of *Yap* in the developing lung are still emerging, but in general, in the conducting airway epithelium, nuclear *Yap* is important for maintenance of the stem cell lineage and basal cell proliferation (14, 15); in the distal lung, shuttling of Yap between the nuclear and cytoplasmic compartments is important in alveolar type 2 (AT2) to alveolar type 1 (AT1) cell differentiation (16, 17).

The goal of this study was to characterize the impact of fetal TO on the lung epithelium. To do so, we first performed a *post-hoc* analysis of cell-specific genes derived from the left lower lobes of fetal rabbits with CDH and/or TO and then validated these findings by histology of the left upper lobes. After identifying basal cells as the most dysregulated cell type, we analyzed the whole lungs of mice in a mouse model of FETO to identify these same changes and identify Hippo/Yap as key transcriptional regulators using a lung epithelial cell-specific knockout approach. We lastly show that the lungs of human neonates with congenital TO have clusters of basal cells in the distal lung that are not present in the lung of control infants.

## Materials and Methods

### Human Tissue Use

Archived human tissues were used with a waiver from the Cincinnati Children's Hospital Institutional Review Board (2016-9641) and from the Vall d'Hebron Research Institute.

### Animal Care and Maintenance

All animal use was approved by the Cincinnati Children's Hospital IACUC (2019-0034). Timed pregnant C57BL/6 mice were housed in a barrier facility with chow and water *ad libitum* and 12-h day/night cycles. Timed pregnant New Zealand white rabbits were purchased from Charles River (Wilmington, MA, USA) and similarly housed.

### Rabbit CDH and TO Model

The fetal rabbit CDH and TO model has been previously described (9). In brief, after anesthesia of the pregnant rabbit, in fetuses, a left-sided diaphragmatic defect was created at embryonic day 25 (E25), TO was performed at E27, and fetuses were collected at E30. The left lower lung was collected and used for RNA sequencing and the left upper lobe collected for histology.

### Mouse TO Model

The transuterine mouse TO model has been previously described (12, 13). Briefly, E16.5 C57BL/6 mice were anesthetized and the fetuses exposed by midline laparotomy. Anterior-facing fetuses had ligation of the trachea and one carotid artery and jugular vein by passing a non-cutting needle with 6–0 silk through the uterine wall and around those structures. Progesterone (2 mg) was administered intramuscularly at E17.5 to prevent spontaneous abortion. At E18.5, pregnant mice were anesthetized, the fetuses collected, and both fetuses and dam sacrificed.

### Conditional *Yap* Deletion

Based on previous studies (15) showing efficient depletion of *Yap* from the airway epithelium, we crossed mice with homozygous floxed *Yap1* exons (B6.*Yap1*^*tm*1.*Dupa*^, *Yap*^*Loxp*/*LoxP*^) with mice heterozygous for *cre* under the control of the *Sonic hedgehog* promoter (B6.*Shh*^*tm*1(*EGFF*/*cre*)*Cjt*^, *Shh*^*cre*^) and a single floxed Yap allele (*Shh*^*cre*^*Yap*^+/Δ^) to create *Shh*^*Cre*^*Yap*^Δ/Δ^ fetuses. Genotyping was performed as previously described (15).

### Tissue Processing

Lung tissue was snap frozen on dry ice and stored at −80°C. The lower left lobe in rabbits and the entire lung in mice were homogenized and the RNA extracted using RNEasy kits (Qiagen, Hilden, Germany). Rabbit lung histology was obtained from frozen left upper lung tissue in optimum cutting temperature (OCT) compound (Tissue-Tek, Torrance, CA, USA) and cryostat sectioning. Entire fetal mouse lungs were fixed in 4% paraformaldehyde in phosphate-buffered saline (PBS), dehydrated, paraffinized, and sections obtained. For lung lysates, frozen lung tissue was homogenized in RIPA buffer.

### Immunofluorescence, Immunohistochemistry, and Image Acquisition

For immunohistochemistry (IHC), mouse lung sections were stained using the antibodies below (Table 1) with appropriate secondary antibodies and the ABC Vectastain kit (Vector Laboratories, Burlingame, CA, USA). For immunofluorescence (IF), rabbit lung sections were stained with the antibodies below and appropriate fluorophore-conjugated secondary antibodies. Brightfield imaging was performed on a Nikon NiE upright microscope with ×10 tiled and ×40 magnification. Immunofluorescent images were obtained using a Nikon SpectraX microscope.

**Table 1:** Primary antibodies.

**Antibody**	**Manufacturer/source (catalog no.)**	**Use**	**Dilution**
NGFR	Abcam (ab8874)	IHC	1:1,000
Yap	Cell Signaling (4912S)	IHC	1:1,000
Phospho-Yap	Cell Signaling (13008S)	IHC	1:1,000
Ki67	Abcam (ab16667)	IHC	1:1,000
Pro-surfactant protein C (guinea pig)	Gift from Dr. Jeffrey Whitsett	IF	1:200
CGRP	Abcam (ab360001)	IF	1:200
Acetylated alpha tubulin	Millipore (MABT868)	IF	1:200
Cytokeratin 14	Abcam (ab7800)	IF	1:200
HopX	Abcam (ab57832)	IF	1:200
Mucin 5AC	Abcam (ab3649)	IF	1:200
Scgb1a1 (guinea pig)	Gift from Dr. Jeffrey Whitsett	IF	1:200
Yap	Cell Signaling (4912S)	IF	1:200
Keratin-5	Abcam (ab53121)	IF	1:200

### Quantitative Image Analysis

For rabbit lung specimens, images of the entire lung lobe at ×10 magnification were generated by tile scanning with autofocusing of each frame at ×10 magnification on a Nikon SpectraX microscope. For each analyte, a negative control image was used to determine an intensity threshold and size thresholds determined based on the marker (nuclear *vs*. cytoplasmic, *vs*. apical membrane). Non-lung material was manually excluded, and the percentage of cells expressing each analyte was determined as nuclei associated with the marker divided by the total nuclei. These percentages were then compared using the Wilcoxon rank-sum test with Kruskal–Wallis *post-hoc* comparisons.

### Western Blot

Mouse lung homogenates were electrophoretically separated and transferred to PVDF membranes and probed for cytokeratin-14 (ab7800, 1:1,000; Abcam, Cambridge, UK) with quantification of total protein using REVERT total protein stain (cat. no. 923-11011; LI-COR, Lincoln, NE, USA).

### Flow Cytometry

E18.5 mouse lungs were collected and a single cell suspension created as previously described (18). Cells were stained for nerve growth factor receptor (NGFR) (ab8874, 1:200; Abcam) and EpCam (47-579180, 1:200; eBioscience, San Diego, CA, USA) antibodies using Zombie Red (423109; BioLegend, San Diego, CA, USA) viability dye for dead cell exclusion on a FACSCanto cell (BD Biosciences, Franklin Lakes, NJ, USA) flow cytometry device and using FlowJo v10.7.1 software for quantification.

### PCR

The quantities of lung cell-specific messenger RNAs (mRNAs) in rabbit lung was quantified by SYBR Green (204143; Qiagen) RT-PCR using the oligonucleotides in Supplementary Table 3. Taqman PCR of mouse lung RNA was performed using Applied Biosystems proprietary primers for connective tissue growth factor (CTGF) (Mm01192932_g1), Trp63 (Mm00495788_m1), and GAPDH (cat. no.4308313; Applied Biosystems, Waltham, MA, USA).

### mRNA-Seq and Bioinformatic Analysis

Lung cell-specific mRNAs were extracted from LungGENS E16.5, E18.5, postnatal day 1 (PND1), PND3, PND7, and PND28 profiles (19). The FKPM (fragments per kilobase of transcript per million mapped reads) values of these genes were extracted from a previously published rabbit CDH and TO dataset (GSE84811). The median FKPM per group was used for the comparison of cell-specific mRNAs. For mouse TO experiments, RNA from control and TO mouse lungs was barcoded using NexteraXT and Illumina oligos and sequenced at a depth of 10 million reads per sample with 150-bp paired-end sequencing on a NovaSeq sequencer (Illumina, San Diego, CA, USA). Reads were aligned to GRCm38 using STAR (20) and count files were imported into DESeq2 [(21), p. 2] using a threshold of five reads per gene in all samples. Differential expression by group was determined. Deferentially expressed genes (DEGs) with an adjusted *p*-value of <0.1 and a fold change of two or greater were exported into ToppGene (22) and Ingenuity Pathway Analysis (IPA) (23) for gene set enrichment analysis (GSEA).

### Statistical Analysis

R version 4.0.2 (24), ggpubr (25), and rstatix (26) were used for graphical and statistical comparisons using one-way ANOVA and Holm–Sidak *post-ho*c test for parametric and Kruskal–Wallis with Dunn's *post-hoc* test for non-parametric comparisons. If comparisons between more than four groups were performed, we applied Bonferroni correction for multiple comparisons. *P* < 0.05 considered significant. Line and whisker plots indicate medians and 25th and 75th percentiles.

## Results

### Increased Numbers of Lung Basal Cells in the Lung Tissue of the Rabbit FETO Model

**Figure 1:**
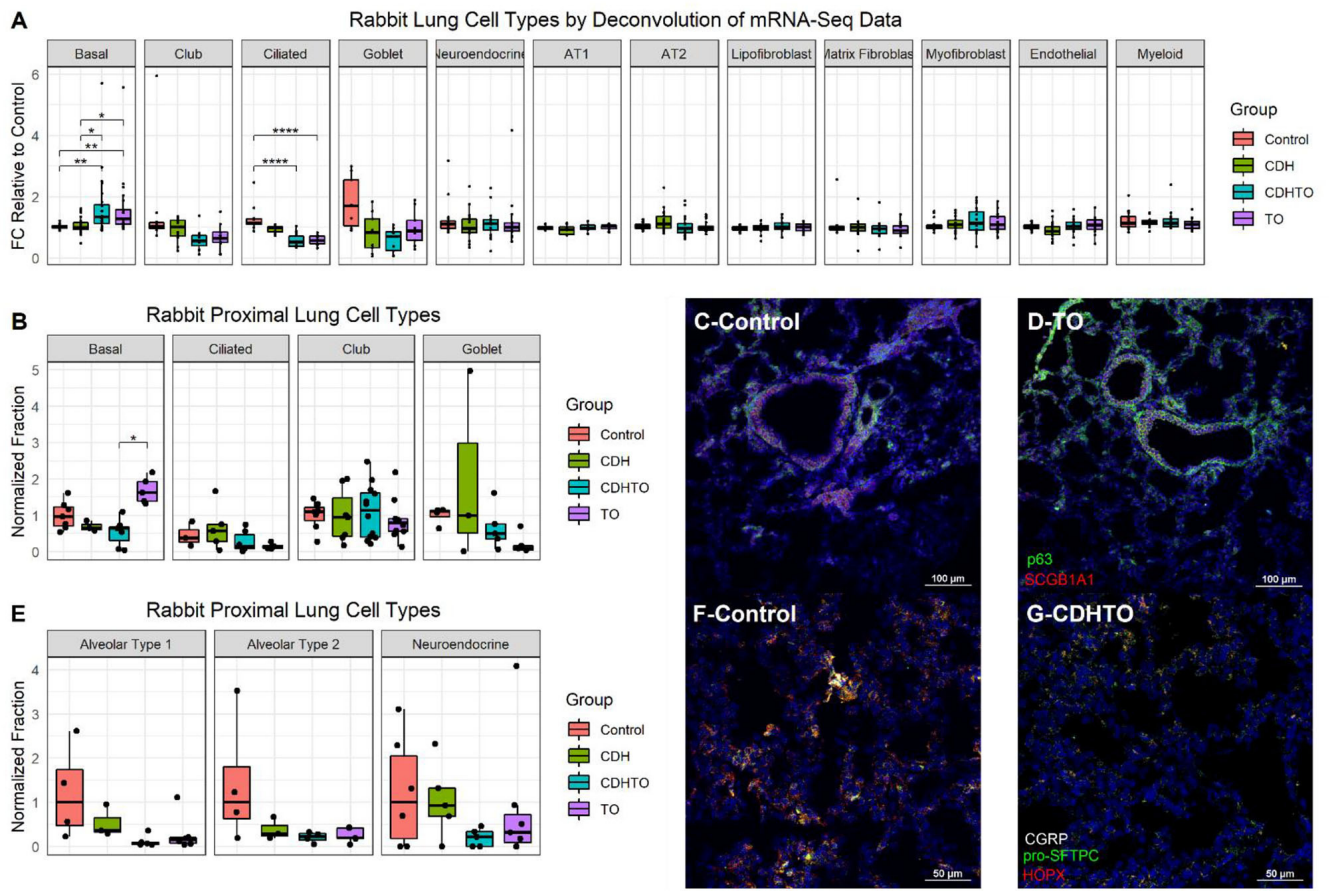
Increased basal cells in a rabbit model of fetal tracheal occlusion. **(A)** Lung cell-specific mRNAs and the relative expression of each in rabbit fetuses with sham surgery, diaphragmatic hernia creation (*CDH*), tracheal occlusion (*TO*), or both (*CDHTO*) were identified. Each data point represents the relative expression of one gene per group (*n* = 4 per group). This analysis identified a relative increase in basal cell- and a reduction in ciliated cell-associated genes. *p < 0.05, **p < 0.01, ****p < 0.0001 by Dunn's *post-hoc* test with Bonferroni correction. **(B)** Immunostaining and quantitative image analysis of the upper left lobe rabbit lung showing a relative increase in the number of basal cells as a fraction of the total cells in TO lung (*n* = 4–6 per group). **(C)** The basal cell master regulator p63 was present in conducting airways of control rabbit fetuses largely below SCGB1A1-positive club cells. **(D)** The relative abundance of p63-positive cells was increased in the lungs of TO rabbit fetuses. **(E)** Apparent reductions of both alveolar type 1 and type 2 cells were not statistically significant. **(F)** Immunostaining for the alveolar type 2 cell marker pro-surfactant protein C (pro-SFTPC) and the alveolar type 1 cell marker HOPX in sham-treated fetal rabbit lung. **(G)** CDHTO lung appeared to have reductions in both markers.

To begin identifying the lung cells most impacted by FETO, we generated lists of cell-specific mRNAs (Supplementary Table 1) using the Lung Gene Expression Analysis database (19). Normalizing the FKPM values of fetal rabbits undergoing fetal creation of diaphragmatic hernia (CDH), FETO, or both (CDH/FETO) to control, we identified increased relative expressions of basal cell-specific mRNAs and decreased relative expressions of club, ciliated, and goblet cell mRNAs in FETO and CDH/FETO lungs. There were no substantial differences in the relative abundances of AT1, AT2, lipofibroblast, matrix fibroblast, myofibroblast, epithelial, or myeloid cell mRNAs (Figure 1A). As the focus of this project was TO, we extracted the 98 genes with adjusted *p* < 0.1 and 1.5-fold increased or decreased expression in control *vs*. TO and performed GSEA. Cell cycle-related processes were enriched and basal cell genes were overrepresented (Table 2). Quantification of cell-specific mRNAs was consistent with these bioinformatic findings (Supplementary Figure 1). On the protein level, cell-specific markers identified an increased number of basal cells in TO lung (Figures 1B–D) and reduced numbers of AT1 cells in CDH, TO, and CDH/TO lung compared to control (Figures 1E–G). This analysis of lung tissue from a previously published rabbit CDH/TO study showed that TO lung has increased numbers of basal cells compared to control.

**Table 2:** Gene set enrichment analysis of fetal rabbit to vs. Control.

**GO molecular function**				**GO biological function**				**Topp cell atlas**			
**Term**	**No. genes**	**% Genes**	**q-value**	**Term**	**No. Genes**	**% Genes**	**q-value**	**Term**	**No. Genes**	**% Genes**	**q-value**
DNA replication origin binding	5	5%	3.65E-05	pre-replicative complex assembly	5	5%	1.34E-10	Cycling Basal Cell (Mouse Trachea)	20	20%	6.43E-24
Calcium ion binding	15	15%	2.68E-03	pre-replicative complex assembly involved in cell cycle DNA replication	5	5%	1.34E-10	Basal Cell (Mouse Trachea)	17	17%	2.83E-19
Glycosaminoglycan binding	8	8%	1.60E-02	pre-replicative complex assembly involved in nuclear cell cycle DNA replication	5	5%	1.34E-10	Dividing T-cell	17	17%	2.09E-18
Extracellular matrix structural constituent	7	7%	1.65E-02	regulation of cell population proliferation	32	33%	3.83E-10	Activated T cell	17	17%	3.55E-18
DNA helicase activity	5	5%	1.73E-02	tube development	26	27%	4.08E-10	Dividing Alveolar Macrophage	16	16%	1.17E-16
Single-stranded DNA binding	6	6%	1.92E-02	circulatory system development	26	27%	6.62E-10	Neuroepithelial cell	16	16%	1.77E-16
3'-5' DNA helicase activity	3	3%	3.88E-02	vasculature development	21	21%	1.73E-09	Developing Plasmablast	16	16%	1.77E-16
Single-stranded DNA helicase activity	3	3%	4.59E-02	double-strand break repair *via* break-induced replication	5	5%	1.86E-09	Early Lung Fibroblast Progenitor	14	14%	5.64E-14
Cell adhesion molecule binding	11	11%	5.59E-02	cell adhesion	27	28%	4.03E-09	AT1-AT2 progenitors	14	14%	2.24E-13
Catalytic activity, acting on DNA	7	7%	5.79E-02	biological adhesion	27	28%	4.44E-09	B-cell	14	14%	2.41E-13

### Increased Number of Basal Cells After Fetal Mouse Tracheal Occlusion

**Figure 2:**
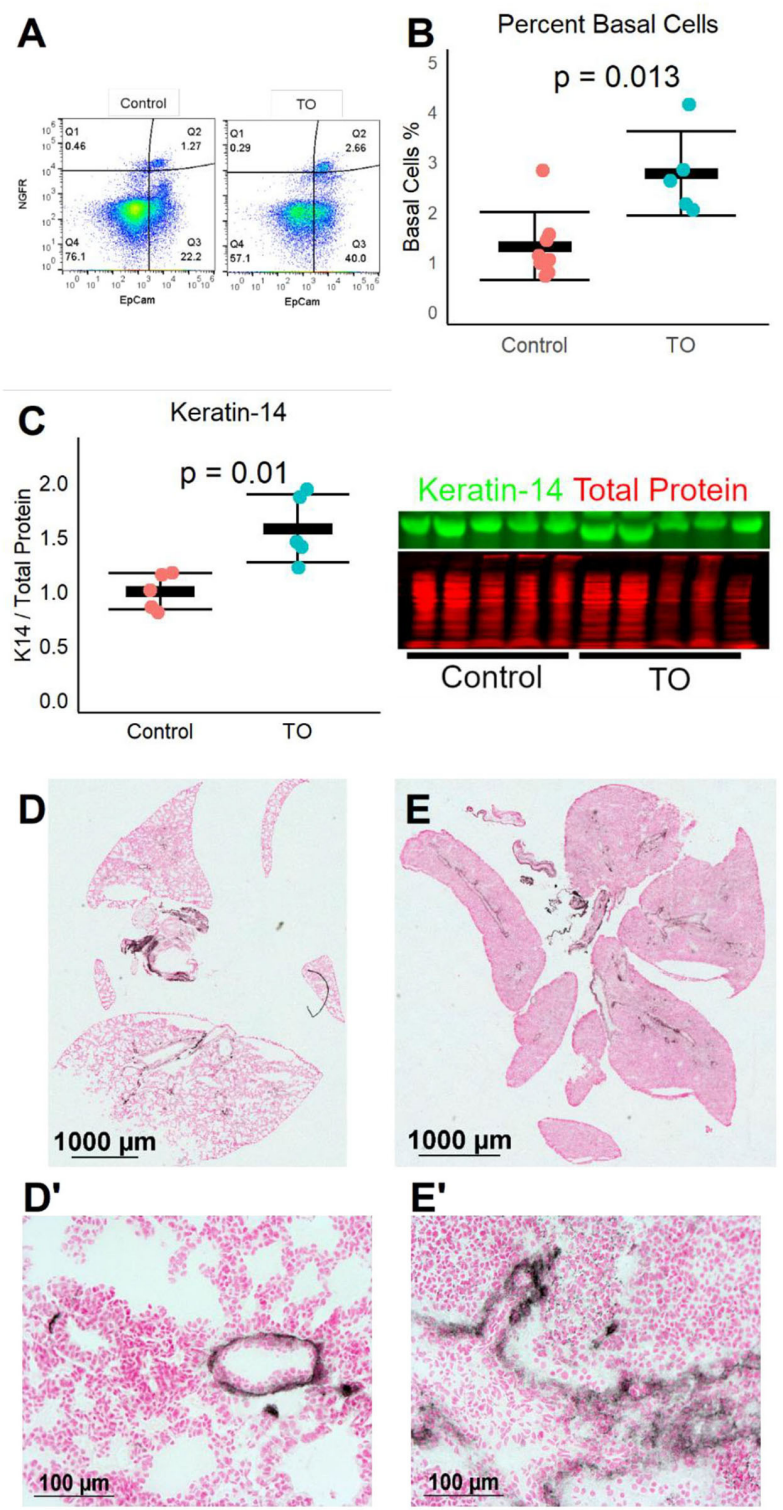
Fetal endoscopic tracheal occlusion (FETO) increases the number and distalization of lung basal cells. **(A)** The percentage of lung basal cells as defined by the co-expression of EpCam and NGFR increased from ~1.5 to ~2.5% at E18.5 following tracheal occlusion (TO) at E16.5. **(B)** The increased percentage of basal cells in TO (*n* = 6) compared to control (*n* = 7) was consistent and statistically significant. **(C)** The whole-lung protein content of the basal cell marker keratin-14 was also increased (*n* = 5 per group). **(D)** Immunohistochemistry of control lung for NGFR showed restriction of basal cells to the basement membrane of large conducting airways. **(E)** FETO lungs had increased cellularity and had extension of basal cells beyond the central conducting airways into the more distal conducting airways. **(D′)**, Magnified Image of D; **(E′)**, Magnified Image of E.

We next used a recently described mouse fetal TO model (12) to test whether the same increase in lung basal cells was seen in a different animal model of TO. After TO at E16.5, E18.5 fetal lungs were collected and single cell suspensions were analyzed by flow cytometry. TO lungs had more than twice the number of basal cells of control lungs (Figures 2A,B). In a separate experiment, the lungs of TO mice had ~2.5-fold greater quantity of the basal cell marker ketatin-14 (Figure 2C). Analysis of whole-lung sections of control and FETO mice revealed increased abundance and more distal extension of basal cells in TO lung compared to control (Figures 2D,E). These fetal mouse data corroborate findings in a fetal rabbit model showing that TO increases the number of basal cells.

### Activation of Yap Signaling by FETO

**Figure 3:**
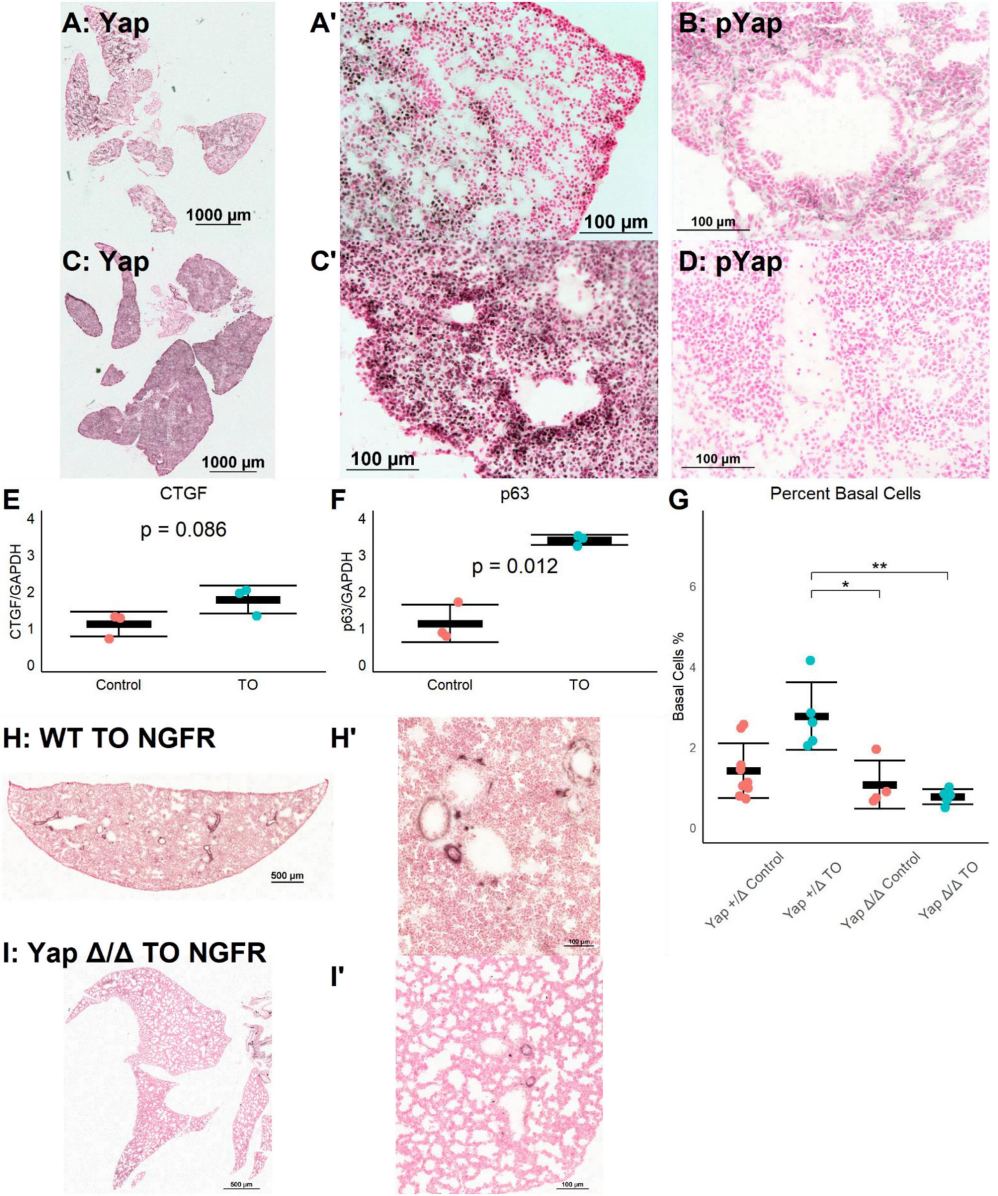
Increased lung epithelial Yap signaling is responsible for basal cell expansion. **(A,B)** In control E18.5 fetuses, non-phosphorylated (nuclear, active) Yap is present in small clusters of distal lung cells **(A)** and phosphorylated (cytosolic, non-active) Yap is widely present **(B)**. **(C)** In E18.5 TO lung, non-phosphorylated Yap is present more homogenously throughout the lung and can be detected more in nuclei than in control, with denser abundance of nuclear-Yap-containing cells round airways. **(D)** In TO lung, phosphorylated Yap is less abundant than in control lung. **(E,F)** PCR of lung homogenates showed increased levels of connective tissue growth factor (*CTGF*) **(E)** and *p63* **(F)**. Both are Yap target genes. Comparison is by Welch's *t*-test. **(G)** Mice with hemizygous deletion of lung epithelial cell Yap at baseline (*n* = 9) and after TO (*n* = 5) showed similar numbers of basal cells to wild-type (WT) mice. Lung epithelial cell-specific deletion of Yap without TO (*n* = 4) or with TO (*n* = 5) both had basal cell frequencies comparable to control hemizygous and WT mice. *p < 0.05, **p < 0.01 by Holm–Sidak *post-hoc* test. **(H,I)** Compared to wild-type **(H)**, mice with conditional deletion of Yap from the lung epithelium **(I)** had reduced abundance of basal cells as assessed by nerve growth factor receptor (NGFR) immunohistochemistry. *Scale bars*, 500 and 100 μm. **(A′)**, Magnified Image of A; **(C′)**, Magnified Image of C; **(H′)**, Magnified Image of H; **(I′)**, Magnified Image of I.

The Yap pathway is both a regulator of epithelial cell proliferation and organ size and a direct regulator of p63, a necessary transcription factor for basal cells (15). Yap signaling is regulated by a series of kinases, with phosphorylated Yap (phospho-Yap) being targeted for degradation and non-phosphorylated Yap translocating to the nucleus where TEAD factors are recruited, activating Yap-regulated genes (27). Thus, non-phosphorylated Yap is transcriptionally active, while phospho-Yap is sequestered in the cytoplasm. The immunohistochemistry for Yap and phospho-Yap identified grouped Yap-positive and phospho-Yap-positive cells in E18.5 lung (Figures 3A,B). In contrast, in E18.5 TO lungs, most nuclei were Yap-positive, and phospho-Yap staining was nearly absent (Figures 3C,D). The mRNA levels of the Yap target genes connective tissue growth factor (*CTGF*) and *Trp63* (*p63*) were both increased in TO lung (Figures 3E,F). These data demonstrate the activation and transcriptional activity of Yap following TO.

To test whether TO activation of Yap was directly responsible for basal cell expansion, we conditionally deleted Yap from the epithelium of fetuses using a Sonic hedgehog driver and floxed Yap alleles. There was a non-significant reduction in the number of basal cells between *Yap*^*LoxP*/*LoxP*^ or *Yap*^+/Δ^ mice and *Yap*^Δ/Δ^ at baseline and a significant reduction in the number of post-TO basal cells in *Yap*^Δ/Δ^ (Figures 3G–I). While these data cannot differentiate whether the lack of TO-induced basal cell expansion is from the absence of a necessary progenitor or the lack of a necessary transcriptional driver, they do show that *Yap* is essential for basal cell expansion in TO.

### Pathways and Processes Impacted by Tracheal Occlusion

**Figure 4:**
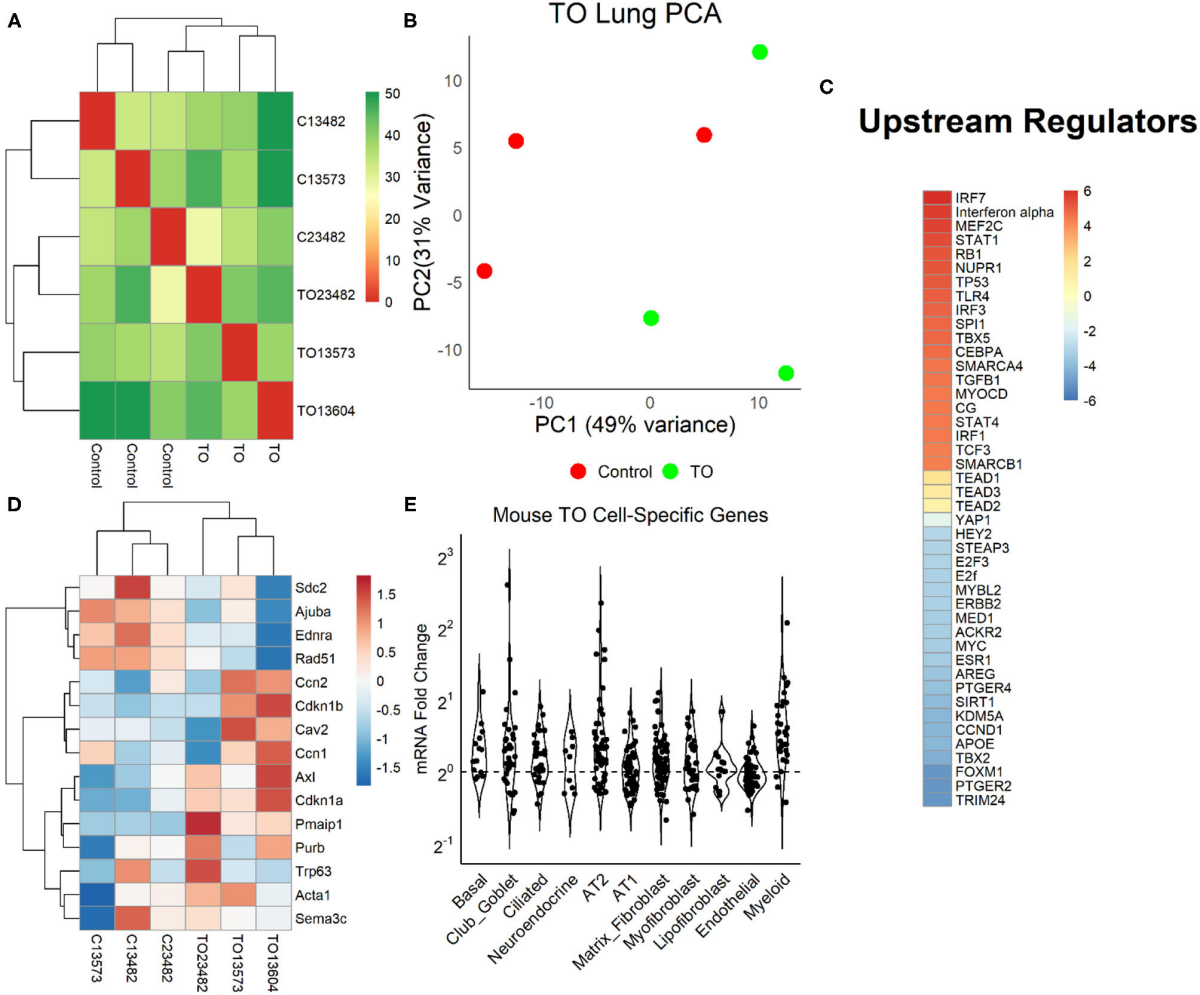
Yap in mouse tracheal occlusion (TO) lung. **(A)** mRNAs from the lungs of three control and three TO fetuses analyzed by mRNA-seq. As assessed by sample distances, TO and control lungs were most similar to one another. **(B)** Principal component plot showing incomplete separation of control from TO specimens. **(C)** Ingenuity Pathway Analysis of upstream regulators of genes that were 2-fold changed between control and TO lungs showing that the regulators with the greatest predicted activation (*red*) were largely related to inflammation and those with the greatest predicted inactivation (*blue*) were mostly related to cell cycle. Yap is transcriptionally inactive when phosphorylated, and non-phosphorylated YAP recruits TEAD factors to response elements. YAP and TEAD factors were not among the top 20 activated and inactivated regulators, but were up- and downregulated consistent with the increased nuclear Yap in TO. **(D)** The mRNA levels of genes known to be regulated by Yap were consistent with the increased nuclear Yap in TO lung specimens. **(E)** Assessment of lung cell-specific mRNAs showing increased basal cell (1.17), club/goblet cell (1.15), AT2 (1.21), and myeloid cell (1.45) mRNAs in TO compared to control lungs.

We examined the relative weight of Yap transcriptional activity compared to other impacted signaling pathways by performing mRNA sequencing (mRNA-seq) and differential gene expression analysis in three control and three TO E18.5 lungs. TO lungs were most similar to each other (Figure 4A), although the separation between control and TO specimens was incomplete when assessed by the first two principal components (Figure 4B). After filtering for genes that contained at least five reads in three or more of the specimens, 17,374 genes were used in the analysis. Among these genes, only 42 had adjusted *p*-values of <0.1. GSEA of these genes identified inflammation-related, tube morphogenesis, and blood vessel morphogenesis processes as differentially regulated (Supplementary Table 2). For further analysis, we analyzed genes that were 2-fold increased (626 genes) or decreased (207 genes). Among the upregulated genes, the biological processes that were activated were cardiac and striated muscle function-related, and there were no significant molecular functions identified among the downregulated genes (Supplementary Figure 2). Upstream analysis of genes with a 2-fold change in expression identified interferons and Toll-like receptors as activated, but it also identified regulators important in cell cycle and lung development, such as *TBX5, SPI1, CEBPA, TGFB1, AREG, FOXM1*, and *CCND1* (Figure 4C). Yap/Taz phosphorylation was predicted to be decreased (activation *z*-score = −0.82, adjusted *p* = 1.49 × 10^−7^) and *TEAD2* activity increased (activation *z*-score = 1.63, adjusted *p* = 2.7 × 10^−7^) in this analysis, but the relative weights of these differences were smaller than those of other regulators. Clustering of specimens using genes known to be regulated by Yap showed that TO and control specimens were distinct from one another (Figure 4D). In performing analysis of lung cell-specific mRNAs similar to that done in fetal rabbit lungs, it was found that basal cells, club/goblet cells, AT2, and myeloid cell mRNAs were increased in TO lung compared to control (Figure 4E).

### Human Tracheal Occlusion Results in Increased Yap and Extension of Basal Cells Distally

**Figure 5:**
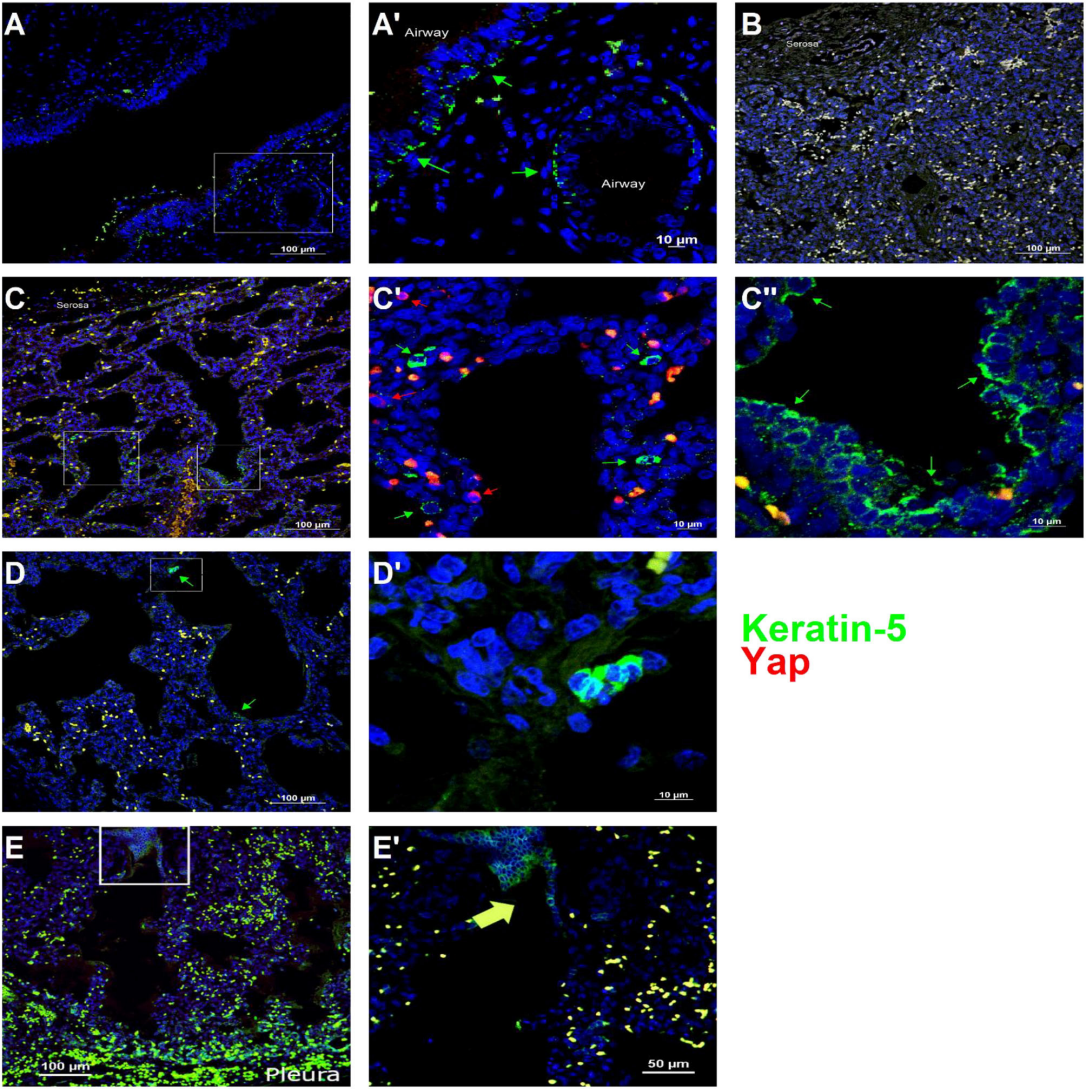
Basal cells are present in the distal lung of infants with congenital high airway obstruction (CHAOS). **(A)** Staining was performed on lung specimens from infants of gestational age 34–36 weeks who died within 48 h of birth with CHAOS, being a human analog to mouse tracheal occlusion. The lungs of both CHAOS and control infants had keratin-5-positive cells (*green arrows*) beneath the pseudostratified epithelium of the large conducting airways. **(B)** The subpleural lung of control specimens had no keratin-5-positive cells. **(C)** CHAOS lung had regions with clusters of keratin-5-positive cells. None of these cells were Yap-positive (*red arrows*), although cells containing both cytoplasmic and nuclear Yap were observed near keratin-5-positive cells. **(D)** Another CHAOS lung specimen showing clusters of keratin-5-positive cells just below the pleura. **(E)** Specimen of an infant with congenital diaphragmatic hernia treated with fetal endoscopic tracheal occlusion who died shortly after birth. In airways just below the pleura, clusters of keratin-5-positive basal cells can be identified. **(A′)**, Magnified Image of A; **(C′)**, Magnified Image of C-box 1; **(C″)**, Magnified Image of C-box 2; **(D′)**, Magnified Image of D; **(E′)**, Magnified Image of E.

To determine whether Yap activation and distal lung basal cell expansion also occurred in human TO, we analyzed lung specimens from two infants who died shortly after birth with congenital high airway obstruction (CHAOS) (gestational ages, 34 and 36 weeks), three infants who died either before or shortly after delivery following FETO for CDH, and two lung specimens of infants who died of non-pulmonary causes at those same ages. All lung specimens had the typical position and spacing of basal cells in the major conducting airways (Figure 5A), and basal cells were not identified apart from these conducting airways in control lung specimens (Figure 5B). CHAOS lung specimens (Figures 5C,D) had clusters of basal cells in the airspaces of the distal lung with Yap-positive nuclei close to these cells. FETO lung specimens (Figures 5E,F) showed expansion of basal cells in the distal airways, although nuclear Yap staining was not prominent. These data are in agreement with the findings in a mouse model of TO with regard to Yap activation and the presence of basal cells in the distal lung airspaces.

## Discussion

This is the first study to show changes in the distribution of lung cell populations in response to FETO and is also the first to study the role of a specific signaling cascade in TO. While the role of Yap in specifying lung epithelial cell populations is well established, this study is the first to show its relevance in human lung development. To the best of our knowledge, this is also the first study using transgenic mice as fetal TO models. To date, animal studies have been hampered by the need to use large animal models that are less amenable to genetic manipulation. The development of a minimally invasive mouse TO model (12) allowed us to use a lung epithelial cell-specific deletion of Yap to mechanistically examine its role in the observed expansion of basal cells in TO mouse lung.

Yap has well-established roles in defining the proximal conducting airway epithelium in early lung development (15) and in maintaining lung basal cells in more mature lung (28). In the distal lung epithelium, cytoplasmic Yap plays an important role in AT2 cell and eventual AT1 cell differentiation. Failure of Yap to undergo nuclear–cytoplasmic shuttling (i.e., sequestered in the nucleus) leads to the disorganization of the conducting airway epithelium without evidence of increased proliferation and with evidence of ectopic AT2 and AT1 cell markers (17). However, Yap has also been recently shown to mediate AT1-to-AT2 cell conversion with an important role in post-injury repair (29). Mst1 and Mst2 phosphorylate Yap, leading to cytoplasmic sequestration. *Mst1* and *Mst2* deletion also impairs the differentiation of conducting airway epithelial cells, but increases the proliferation of these undifferentiated epithelial cells (14). In the study of Lange et al. increased total and nuclear Yap led to the expansion of lung basal cells at the expense of club cells. We did not observe any increases in AT1, as previously described in mice with deletions of *Lats1* and *Lats2* (which are downstream of *Mst1* and *Mst2*, also leading to increased nuclear Yap) and in a recent study of *ex vivo* inflation of fetal mouse lungs (30). Interestingly, ectopic p63-positive cells were observed in *Mst1*- and *Mst2*-deficient mice (16). Our finding that a TO-mediated increase in nuclear Yap mediates the expansion of the lung basal cell population is largely consistent with the literature on the role of Yap in lung epithelial cell differentiation and function, with discrepant findings likely attributable to the differences in the five models (constitutive nuclear Yap, *Mst1* and *Mst2* deletion, *Lats1* and *Lats2* deletion, *ex vivo* lung inflation, and TO with lung epithelial cell Yap deletion).

Clinically, little is known about the post-FETO lungs of infants with CDH. While FETO improves the survival of fetuses with the most severe forms of CDH (1), impaired lung function is a substantial problem in survivors. The importance of basal cell clusters in the distal lung is also unclear. Basal cells or basal cell progenitors have been shown to be contributors to distal lung regeneration following influenza infection (28), and keratin-14-positive cell “pods” can be identified in normal and regenerating adult distal lungs (31). Increased percentages of basal cells are associated with several non-malignant pulmonary diseases when assessed by single-cell mRNA-seq (32). Our studies are not comprehensive enough to describe when keratin-14-positive pods may appear in the human lung as we did not observe any in control lung specimens. Whether these cells persist or are adaptive or deleterious in the context of subsequent lung injury can now be assessed by removal of the tracheal ligature at E17.5 with subsequent parturition and raising of post-TO mice (33).

Although we focused on Yap and basal cells, our bioinformatic data suggest that other signaling cascades are more significantly impacted than this one. The most notable difference between TO and control mouse lungs was the increased inflammation in TO lung. The role of different lung resident macrophages in lung development is being increasingly appreciated (34), and how TO alters the relative abundance and activity of these populations is likely important in post-TO lung. TO lung also has activation of several cardiac and skeletal muscle-related pathways. This could be from three non-exclusive sources: lung myofibroblasts, vascular smooth muscle cells, and airway smooth muscle cells. Lung mesenchymal cells in general and lung myofibroblasts in particular play an important role in saccular and alveolar lung development (35, 36). Pulmonary vascular pruning, vascular smooth muscle hypertrophy, and pulmonary hypertension are substantial problems for infants with CDH (2). In rabbit models of CDH and TO, TO enhanced lung perfusion (37) and reduced arterial wall thickness after surgical diaphragmatic hernia (38). The potential impact of TO in airway smooth muscle hypertrophy and lung fibroblast populations merits further investigation, which is now more achievable with mouse TO models. Furthermore, TGF-β has been consistently reported as increased after TO in rabbit and ovine models (7, 9, 10), and our study shows this to be true in the mouse model as well. While caution is required when making cross-species comparisons (9), consistent findings across multiple studies should provide confidence that the observed alterations are likely operative in humans.

Several study limitations are noted. For the quantification of the lung cell populations in fetal rabbit lungs, quantitative image analysis of lung sections is subject to measurement errors due to differences in tissue sections, sectioning plane, and imaging plane. The staining of all sections together and imaging, thresholding, and quantification using identical settings somewhat mitigate these concerns, and the correlation of image analysis and mRNA data provides confidence with regard to the relative changes in cell populations between experimental groups. However, the comparison of the relative abundance of one cell population to another should be made with caution. We quantified only the relative percentage of basal cells in TO lungs. This is relevant because hypercellularity is known to occur following TO (9, 12). We did not determine whether there were increased quantities of basal cell-associated mRNAs and proteins in TO basal cells, or if this increased expression was due solely to an increased number of basal cells. We did not explore the impact of TO on mesenchymal or immune cells, although these signatures were more highly altered than that of Yap and basal cells. Of particular note, we did not deeply investigate or differentiate changes in gene expression that may have arisen from the pulmonary vasculature, which is known to be impacted by both CDH and TO (38). Finally, we could not obtain any lung tissues to directly assess basal cells in post-TO human lung, but we were able to secure a small number of specimens from infants with congenital occlusion of the trachea. Development of a biobank for CDH and FETO-treated lung specimens of expired infants would greatly enhance the translatability of animal work being done in this area.

In summary, we have shown that Yap mediates the expansion of the basal cell population in mouse and human lungs in response to tracheal occlusion.

## Data Availability Statement

The datasets presented in this study can be found in online repositories. The names of the repository/repositories and accession number(s) can be found at: GSE184865, https://www-ncbi-nlm-nih-gov.ezproxy.u-pec.fr/geo/query/acc.cgi?acc=GSE184865.

## Ethics Statement

The animal study was reviewed and approved by Cincinnati Children's Hospital Medical Center, IACUC 2019-0034.

## Author Contributions

VS, CS, RJ, EA, and BV contributed to the conception and design of the study. EA, MO, MM, and JP all developed the assays and surgical techniques and identified clinical specimens required for the study. BV wrote the first draft of the manuscript. All authors contributed to manuscript revision, read, and approved the submitted version.

## Funding

Funding was obtained from NIH/NHLBI R01HL141229 (to BV).

## Conflict of Interest

The authors declare that the research was conducted in the absence of any commercial or financial relationships that could be construed as a potential conflict of interest.

## Publisher's Note

All claims expressed in this article are solely those of the authors and do not necessarily represent those of their affiliated organizations, or those of the publisher, the editors and the reviewers. Any product that may be evaluated in this article, or claim that may be made by its manufacturer, is not guaranteed or endorsed by the publisher.
