# Supplementary material for: Fetal Tracheal Occlusion Increases Lung Basal Cells via Increased Yap Signaling
Source: Front Pediatr. 2022 Feb 22;9:780166. doi: 10.3389/fped.2021.780166 (PMC8904268; doi:10.3389/fped.2021.780166)
Supplement: Supplementary file 1 [file Presentation_1.pdf]

Fetal Tracheal Occlusion Increases Lung Basal Cells via Increased Yap Signaling

SUPPLEMENTAL MATERIAL

PCR Validation of Fetal Rabbit Lung mRNA-Seq Cell-Specific mRNAs

We quantified the mRNA levels of several cell-specific mRNAs in Control, CDH, TO, and CDHTO fetal rabbit lung. In general, the data showed good correlation of the cell-specific mRNAs with one another, a reduction in ciliated cell mRNAs, and increased basal cell and alveolar type 1 cell mRNAs (Supplemental Figure 1).

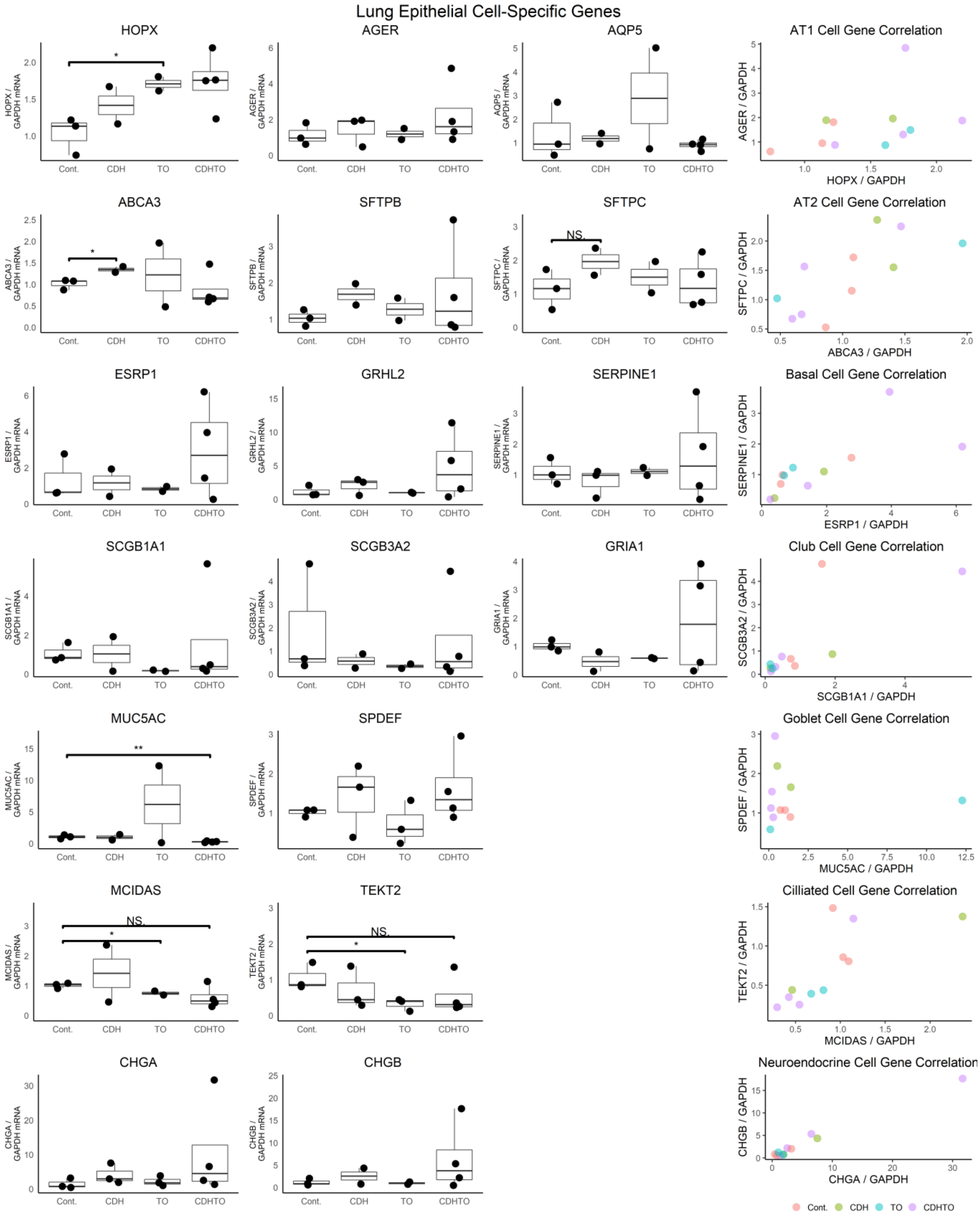
Lung Epithelial Cell-Specific Genes
HOPX
AGER
AQP5
AT1 Cell Gene Correlation
ABCA3
SFTPB
SFTPC
AT2 Cell Gene Correlation
ESRP1
GRHL2
SERPINE1
Basal Cell Gene Correlation
SCGB1A1
SCGB3A2
GRIA1
Club Cell Gene Correlation
MUC5AC
SPDEF
Goblet Cell Gene Correlation
MCIDAS
TEKT2
Cilliated Cell Gene Correlation
CHGA
CHGB
Neuroendocrine Cell Gene Correlation
HOPX / GAPDH mRNA
AGER / GAPDH mRNA
AQP5 / GAPDH mRNA
ABCA3 / GAPDH mRNA
SFTPB / GAPDH mRNA
SFTPC / GAPDH mRNA
ESRP1 / GAPDH mRNA
GRHL2 / GAPDH mRNA
SERPINE1 / GAPDH mRNA
SCGB1A1 / GAPDH mRNA
SCGB3A2 / GAPDH mRNA
GRIA1 / GAPDH mRNA
MUC5AC / GAPDH mRNA
SPDEF / GAPDH mRNA
MCIDAS / GAPDH mRNA
TEKT2 / GAPDH mRNA
CHGA / GAPDH mRNA
CHGB / GAPDH mRNA
Cont.
CDH
TO
CDHTO
*
**
NS.
HOPX / GAPDH
AGER / GAPDH
ABCA3 / GAPDH
SFTPC / GAPDH
ESRP1 / GAPDH
SERPINE1 / GAPDH
SCGB1A1 / GAPDH
SCGB3A2 / GAPDH
MUC5AC / GAPDH
SPDEF / GAPDH
MCIDAS / GAPDH
TEKT2 / GAPDH
CHGA / GAPDH
CHGB / GAPDH
Cont. CDH TO CDHTO

Supplemental Figure 1: PCR quantification of cell-specific mRNAs in Fetal Rabbit lung. PCR for the AT1 cell markers Homeodomain-only protein homeobox (HOPX), Advanced Glycosylation End-products Receptor (AGER), and Aquaporin 5 (AQP5), the AT2 cell markers ATP Binding Cassette Subfamily A Member 3 (ABCA3), Surfactant Protein B (SFTPB), and SFTPC, the basal cell markers Epithelial Splicing Regulatory Protein 1 (ESRP1), Grainyhead Like Transcription Factor 2 (GHRL2), and Serpin Family E Member 1 (SERPINE1), the club cell markers Secretoglobin Family 1A Member 1 (SCGB1A1), SCGB3A2, and Glutamate Ionotropic Receptor AMPA Type Subunit 1 (GRIA1), the ciliated cell markers Multiciliate Differentiation And DNA Synthesis Associated Cell Cycle Protein (MCIDAS), and Tektin 2 (TEKT2), and the neuroendocrine cell markers Chromogranin A (CHGA) and CHGB were generally consistent with mRNA-seq data showing increased AT1 and basal cell mRNAs and decreased ciliated cell mRNAs.

Cell-specific Genes

Genes in Supplemental Table 1 were used for deconvolution of bulk mRNA-seq data.

Supplemental Table 1: Cell-Specific Genes

AT2	AT1	Basal	Ciliated	Club	Neuroendocrine	Endothelial	Myeloid	Matrix Fibroblast	Myofibroblast	Lipofibroblast
Egfl6	Tspan8	Igf1	3300002A11Rik	Gm15883	Crcp	Eltd1	Ms4A6D	Col3A1	Kcnj8	Adh1
Pla2G1b	Ctgf	Itga6	Lrrc71	Lrrc26	Ngfrap1	Aplnr	Clec4A3	Mxra8	Enpp2	Col12A1
Lgi3	Tinagl1	Snai2	1700016K19Rik	Cckar	Ngfr	Plvap	Ai607873	Col1A1	Gpr64	Slc36A2
Lamp3	Mal2	Nrg1	Bc051019	Gpx2	Chga	Emcn	Fpr2	Cped1	Pdgfra	Slc46A2
Scd1	Wfdc1	Il13Ra2	Tekt2	Lypd2	Chgb	Ripply3	Sirpb1B	Fibin	Dnm3Os	Fhl1
Atp6v2c2	Lamc2	Ezh2	1700003M02Rik	Krt15	Pyy	Sox17	Ccr1	Vldlr	Agt	Shisa2
Hc	Ndst1	Tp63	1700012B09Rik	Cyp2F2	Sypl1	Clec1A	Clec4N	Gpc3	Hlf	Aars2
Slc26slC9a9	Phactr1	Grhl2	D430036J16Rik	Gsta3	Edn1	Ushbp1	Sirpb1C	Col6A2	Tbx5	Ppp1R3F
Rbpjl	Gja1	Stat3	Stk33	Ehf	Calcrl	Clec14A	C5Ar1	Serpine2	H19	Col25A1
Chi3chI1l1	Cryab	Ngfr	4833427G06Rik	Homer2	Robo1	Kit	Spi1	Cp	Pdzrn3	Fancc
Spink5	Ano1	Tubb6	1700026D08Rik	Kcnk2	Robo2	Tek	Fcgr3	Fblim1	Rhou	Thg1L
Bex2	Lmo7	Krt14	Nek5	Gsta4	Robo3	Myzap	Clec4A2	Palld	Fibin	Hebp1
Elf5	Scnn1A	Scel	Nme5	Ace2	Robo4	Icam2	Ncf2	Snai2	Hhip	Lipe
Ppp1R14c	Sphk1	Snai2	Dnaaf1	Aox3	Slit1	Rasip1	Ms4A6C	Col13A1	Prdm6	Thap2
Tfcp2tfcp1l1	Krt8	Serpine2	Ttll6	Aldh1A7	Slit2	Arhgef15	Mpeg1	Rbp1	Tgfbi	Mvk
Cxcl15	Il18R1	Thbs1	Ccdc147	B430010I23Rik	Slit3	Tie1	Cd300Lf	Cdo1	Igf1	Enpep
Kcnc3	Fam189a2	Dlk2	1700001L19Rik	5330417C22Rik		Jam2	Fcgr2B	Dpep1	Dkk3	Rras2
Dram1	Flrt3	Bcam	Elmod1	Aldh1A1		Cdh5	Wfdc17	Ppp1R14A	Crispld2	Chst15
Muc1	Mthfd1	Abi3Bp	Tekt4	Cxcl17		Myct1	Cd84	Plxdc2	Tbx4	Hoxb3
Etv5	Tspan15	Lamb3	Efcab1	Acsl1		Stmn2	Ifi204	Gyg	Ism1	Kctd5
S100g	Slco3a1	Trp63	Ccdc37	Ptgr1		Prx	Igsf6	Angpt1	Tgfbr3	Sprtn
Tspan11	Fads3	Cald1	Ccdc96	Dapk2		Arap3	Pirb	Echdc2	Gm13889	Thy1
Cebpa	Hopx	Fscn1	Kcnmb2	Tacstd2		Cd93	Tyrobp	Maf	Mdk	Plin2
Retnla	Vegfa	Adamts1	Rsph10B	Cldn10		Adcy4	Il1B	Adarb1	Loxl2	
Bex1	Ppm1l	Plau	Ak7	Acss1		Flt1	Pld4	Loxl1	Enpp2	
Myh7	Ager	Serpine1	Meig1	Rassf9		Ecscr	Ab124611	Nexn	Tnc	
Kcnj15	F3	Stc2	Ccdc135	Synpo		Gata2	Ms4A6B	Fxyd1	Agt	
Fabp5	Krt18	Cav1	4930451C15Rik	Au021092		Ppp1R16B	Laptm5	Fmo2	Loxl2	
Irx3	Wwc1	Upp1	Cdhr3	Fam46C		Rasgrp3	Fcer1G	Gpx3	Rhou	
Irx1	Pdgfa	Lipg	Tcte1	Scgb3A2		Ctla2B	Ctss	Angptl4	Crispld2	
Abca3	Cystm1	Aldh1L2	Map3K19	Acss2		Ptprb	Arhgap30	Plac9A	Tgfbi	
Matn4	Icam1	Psat1	Zbbx	Gss		Fgd5	Ccr2	Plac9B	Pdzrn3	
Fasn	Rtkn2	Phlda1	Fam47E	Wfdc1		Ace	Alox5Ap	Adamts2	H19	
Rmdn2	2210011c24Rik	Il1Rn	Akap14	Stx19		Cldn5	Apbb1Ip	Ednra	Col27A1	
Wfdc2	Msln	Slc16A1	Lrrc48	Trf		Gpihbp1	Csf2Rb	Nebl	Col5A1	
Bex4	Gipc2	Slc7A5	Zfp474	Retnla		S1Pr1	Cybb	Ptprd	Net1	
Ank3	S100a14		1700028P14Rik	Scnn1B		Pecam1	Lilrb4	Pcolce2	Hhip	
Slc34slC2a2	Scnn1g		Ccdc121	Gsto1		Calcrl	Hcls1	Meox2	Gm13889	
Dlk1	Spock2		Efcab10	Cgn		Tspan18	Plek	Hsd11B1	Ism1	
Myo5myO5Cc	Pdpn		2410004P03Rik	Rab25		Pcdh17	Lst1	6030408B16Rik	Tagln	
Lpcat1	Slc6a14		Sntn	Cd55		Ly6A	Pla2G7	Clec3B	Tpm2	
Irx2	Aqp5		Fank1	Fam13A		Ctla2A	Ptprc	Adh1	Dkk3	
Pi4K2b	Col4a3		Agr3	Gstt3		Egfl7	Slfn2	Tcf21	Mdk	
Tc2tc2Nn	Akap5		1600029I14Rik			Erg	Ly86	Spon1	Prdm6	
Dcxr	Sema3a		Fam183B			Palmd	Ccl6	Itga8	Tgfbr3	
Ces1ceS1Dd	Sema3E		Gm867			Tspan7	Gp49A	Cacna1D	Fibin	
Tmem243	Sec14l3		Pifo			Acer2	Snx10	Lbh	Samd4	
Lcn2	Col4a4		Lrrc6			Cyyr1	Tnfaip2	Enpep	Tnfrsf12A	
Fgfr2	Tmem37		Ttc18			Acvrl1	Mnda	Olfml3	Pamr1	
Sftpb	Scnn1B		1700001C02Rik			Kdr	Ai662270	Cdh11	Thbs1	
Prr15prR15Ll	2310007B03Rik		1110017D15Rik			Eng	Ncf1	Ogn	Sdc2	
Sfta2	Cdkn2B		Iqca			Podxl	Gda	Atp1A2	Aspn	
Sftpd	Krt19					Sema6A	Samsn1	G0S2	Tbx5	
Rbm47	Krt7					Nsg1	Cfp	Dpt	Pdlim3	
Sdc1	Pard6B					Nostrin	Sirpa	Mfap4	Spon2	
Cbr2	Gramd2					Esam	2010005H15Rik	Serping1	Fndc1	
Cldn3	Slc39a8					Ahr	Bcl2A1D	Dcn	Gpr64	
Nkx2-1	2200002d01Rik					Hyal2	Bcl2A1A	Mfap5	Olfml2B	
Napsa	Rab11fip1					Ldb2	Bcl2A1B	C1S	Igf1	
Baiap2l1	Tacstd2					Edn1	Csf2Ra	Thbs2	Des	
Snx25	Eps8L2					2810025M15Rik	1810033B17Rik	Ptn	Kcnj8	
Exosc7	Fam174B					Rgs12	Cd300A	Prrx1	Wnt5A	
Acsl4	Llgl2					Tmem2		Fbln1	Dnm3Os	
Lrrk2	Mmp11					Efnb2		C1Rb	Rp23-103I12.13	
Itga9	Gprc5a					Ramp2		Cygb	Ltbp2	
Slc15slC2a2	Myh14					Scn7A		Col14A1		
Sftpa1	Clic3					Impdh1		Pcolce		
Rab27raB27Bb	Pkp2					Mcam		Selm		
Acot1								C1Ra		
Gclc								Igfbp5		
								Col6A3		
								Rcn3		
								Nkain4		
								Col5A1		
								Kdelr3		
								Aebp1		
								Fbn2		
								Postn		
								Aspn		
								Gas1		
								Adamts2		
								Loxl1		
								Ccdc80		
								Bicc1		
								Leprel2		
								Plagl1		
								Sfrp1		
								Lpar1		
								Gpc3		
								Igf1		
								Fbn1		
								Ltbp4		
								Lrrc17		
								Col5A2		
								Rarres2		
								Igfbp6		
								Pamr1		
								Col1A1		
								Loxl2		
								Rian		
								Ogn		
								Nbl1		
								Serping1		
								C1Qtnf7		
								Col3A1		
								Col6A2		
								Smim1		
								Dnm3Os		
								Bgn		
								Clec3B		
								Plxdc2		
								Cdh11		
								Ednra		

Mouse TO mRNA-Seq Gene Set Enrichment Analysis

Significantly different, 2-fold upregulated, and 2-fold downregulated DEGs were analyzed in ToppGene to identify key pathways and processes as described in Supplemental Figure 2.

Supplemental Figure 2: Gene Set Enrichment Analysis of DEGs in Mouse TO

Signficant DEGs

GO Biological Process	q-value
neutrophil degranulation	0.00006978
neutrophil activation involved in immune response	0.00007316
neutrophil activation	0.00008676
neutrophil mediated immunity	0.00009223
granulocyte activation	0.00009507
leukocyte degranulation	0.0001654
myeloid cell activation involved in immune response	0.0001927
myeloid leukocyte mediated immunity	0.0002237
myeloid leukocyte activation	0.0009075
leukocyte activation involved in immune response	0.001529
cell activation involved in immune response	0.001593
regulated exocytosis	0.00416
leukocyte mediated immunity	0.008235
exocytosis	0.01084
exocytic process	0.01084
vesicle fusion to plasma membrane	0.01084
cellular transition metal ion homeostasis	0.01294
immune effector process	0.01477
vesicle fusion	0.01576
organelle membrane fusion	0.01646
organelle fusion	0.01939
leukocyte activation	0.02023
vesicle organization	0.02117
secretion	0.027
transition metal ion homeostasis	0.02762
defense response	0.029
membrane fusion	0.03223
cell activation	0.05176
cellular iron ion homeostasis	0.08439
response to inorganic substance	0.09709

Pathway	q-value
Metal sequestration by antimicrobial proteins	0.00002271
Neutrophil degranulation	0.0001462
IL-17 signaling pathway	0.004126
Interleukin-4 and 13 signaling	0.009228
Innate Immune System	0.02789

DEGs Down

GO Cellular Component	q-value
neuronal cell body membrane	0.06553
cell body membrane	0.07114
fibrinogen complex	0.08099

DEGs Up

GO Biological Process	q-value
myofibril assembly	5.943E-28
striated muscle cell differentiation	6.894E-26
striated muscle cell development	5.428E-25
muscle structure development	2.806E-24
sarcomere organization	2.997E-24
muscle cell development	8.5E-24
cellular component assembly involved in morphogenesis	2.549E-22
striated muscle tissue development	7.775E-22
muscle cell differentiation	1.167E-21
muscle tissue development	6.718E-21
muscle organ development	6.369E-19
muscle system process	2.891E-17
cardiac muscle tissue development	4.299E-17
heart contraction	1.08E-16
striated muscle contraction	1.202E-16
heart process	2.949E-16
muscle contraction	7.248E-16
cardiac muscle tissue morphogenesis	1.779E-15
muscle tissue morphogenesis	4.389E-15
actomyosin structure organization	4.706E-15
blood circulation	4.935E-15
circulatory system process	9.09E-15
actin-mediated cell contraction	1.231E-14
muscle filament sliding	2.848E-14
muscle organ morphogenesis	3.122E-14
actin-myosin filament sliding	4.593E-14
heart development	2.082E-13
cardiac muscle contraction	2.948E-13
circulatory system development	3.378E-13
regulation of heart contraction	6.474E-13
actin filament-based movement	8.983E-13
cardiac muscle cell development	9.388E-13
actin filament-based process	3.151E-12
cardiac cell development	4.183E-12
anatomical structure formation involved in morphogenesis	7.072E-11
cardiac myofibril assembly	9.135E-11
supramolecular fiber organization	9.901E-11
cardiac muscle cell differentiation	2.836E-10
regulation of blood circulation	3.158E-10
cardiocyte differentiation	8.63E-10
heart morphogenesis	1.03E-09
regulation of system process	1.521E-09
skeletal muscle thin filament assembly	2.142E-09
cellular component morphogenesis	2.19E-09
muscle fiber development	6.245E-09
skeletal myofibril assembly	2.476E-08
ventricular cardiac muscle tissue morphogenesis	3.995E-08
regulation of striated muscle contraction	5.251E-08
cardiac ventricle morphogenesis	7.054E-08
ventricular cardiac muscle tissue development	3.555E-07
regulation of muscle system process	4.677E-07
metal ion transport	5.039E-07
skeletal muscle tissue development	6.452E-07
actin cytoskeleton organization	7.917E-07
animal organ morphogenesis	8.315E-07
potassium ion transmembrane transport	9.902E-07
secretion	0.00000116
export from cell	1.458E-06
striated muscle adaptation	1.512E-06
skeletal muscle organ development	1.772E-06
potassium ion transport	1.784E-06
skeletal muscle adaptation	1.849E-06
cardiac muscle fiber development	2.391E-06
secretion by cell	2.874E-06
muscle adaptation	4.799E-06
cardiac chamber morphogenesis	5.919E-06
cardiac ventricle development	7.365E-06
cardiac conduction	0.00001015
cardiac chamber development	0.00001606
regulation of muscle contraction	0.00001751
myotube differentiation	0.00002935
response to other organism	0.00004261
response to external biotic stimulus	0.00004467
peptide secretion	0.00004758
positive regulation of developmental process	0.00008194
establishment of protein localization to extracellular region	0.00008379
response to biotic stimulus	0.00008385
protein localization to extracellular region	0.0001088
regulation of heart rate	0.0001128
regulation of ion transmembrane transport	0.0001317
tissue morphogenesis	0.0001481
striated muscle myosin thick filament assembly	0.0001641
skeletal muscle myosin thick filament assembly	0.0001641
response to bacterium	0.0001766
regulation of transmembrane transport	0.0001977
positive regulation of transport	0.0002538
protein secretion	0.0002593
regulation of ion transport	0.0002721
myosin filament assembly	0.0002999
defense response	0.0003085
response to muscle stretch	0.0003296
myeloid leukocyte migration	0.0004314
regulation of actin filament-based movement	0.000496
regulation of the force of heart contraction	0.0005954
detection of muscle stretch	0.0005972
myosin filament organization	0.0008478
inorganic cation transmembrane transport	0.0009949
skeletal muscle contraction	0.001031
multicellular organismal signaling	0.001038
cation transport	0.001759
inorganic ion transmembrane transport	0.001839
actin filament organization	0.002312
biological adhesion	0.002464
regulation of peptide secretion	0.002637
negative regulation of cation transmembrane transport	0.00312
regulation of secretion	0.003125
leukocyte chemotaxis	0.003459
regulation of cardiac muscle contraction	0.003787
regulation of myotube differentiation	0.004053
adult heart development	0.004317
regulation of cardiac conduction	0.00462
ion transport	0.005222
regulation of protein secretion	0.006173
cation transmembrane transport	0.006283
positive regulation of myotube differentiation	0.006366
regulation of cation channel activity	0.006403
regulation of membrane potential	0.007054
regulation of immune system process	0.008005
regulation of secretion by cell	0.009363
cell adhesion	0.01065
regulation of transporter activity	0.01199
regulation of potassium ion transmembrane transport	0.01226
cell activation	0.01276
negative regulation of ion transmembrane transport	0.01295
inflammatory response	0.01346
regulation of potassium ion transport	0.01412
regulation of striated muscle cell differentiation	0.01434
musculoskeletal movement	0.0149
multicellular organismal movement	0.0149
apoptotic process involved in heart morphogenesis	0.01963
positive regulation of cation channel activity	0.02129
regulation of developmental growth	0.02325
regulation of cation transmembrane transport	0.0237
regulation of response to external stimulus	0.02596
positive regulation of striated muscle cell differentiation	0.0291
negative regulation of transmembrane transport	0.03019
regulation of growth	0.03151
striated muscle hypertrophy	0.03219
neurofilament bundle assembly	0.0326
sarcomerogenesis	0.0326
regulation of metal ion transport	0.03354
chemical homeostasis	0.03638
positive regulation of ion transport	0.03711
muscle hypertrophy	0.03761
neutrophil migration	0.0406
transmembrane transport	0.04336
neutrophil chemotaxis	0.04424
regulation of potassium ion transmembrane transporter activity	0.04452
leukocyte activation	0.04556
positive regulation of immune system process	0.04671
granulocyte chemotaxis	0.05087
monovalent inorganic cation transport	0.05285
regulation of ion transmembrane transporter activity	0.05584
granulocyte migration	0.05605
cation homeostasis	0.05865
myeloid leukocyte activation	0.05905
response to mechanical stimulus	0.07529
immune effector process	0.07578
ion homeostasis	0.07613
regulation of transmembrane transporter activity	0.08006
inorganic ion homeostasis	0.08108
calcium ion transport	0.08537
cellular divalent inorganic cation homeostasis	0.08692

Pathway	q-value
Muscle contraction	5.483E-12
Striated Muscle Contraction	4.611E-11
Diltiazem Pathway	1.222E-06
Verapamil Pathway	1.222E-06
Dilated cardiomyopathy	3.202E-06
Dilated cardiomyopathy	3.202E-06
Hypertrophic cardiomyopathy (HCM)	0.0000111
Hypertrophic cardiomyopathy (HCM)	0.0000111
Cardiac muscle contraction	0.0004838
Cardiac muscle contraction	0.0004838
Metal sequestration by antimicrobial proteins	0.002956
Cardiac conduction	0.006476
Oxprenolol Pathway	0.02019
Nadolol Pathway	0.02019
Pindolol Pathway	0.02019
Penbutolol Pathway	0.02019
Propranolol Pathway	0.02019
Nebivolol Pathway	0.02019
Bisoprolol Pathway	0.02019
Metoprolol Pathway	0.02019
Esmolol Pathway	0.02019
Acebutolol Pathway	0.02019
Atenolol Pathway	0.02019
Alprenolol Pathway	0.02019
Betaxolol Pathway	0.02019
Potassium Channels	0.03818
Adrenergic signaling in cardiomyocytes	0.03901
Labetalol Pathway	0.06223
Carvedilol Pathway	0.06223
Interferon alpha/beta signaling	0.08458

Primers Used for Cell-Specific mRNAs in Rabbit Lung

The primers in Supplemental Table 3 were used with SybrGreen RT-PCR to quantify cell-specific mRNAs.

Supplemental Table 3: Primers for Quantification of Rabbit Lung-Cell Specific mRNAs

Primer	Target	Orientation	Sequence
rGAPDH(812-1283)mRNA-F	GAPDH	Forward	CCTGGAGAAAGCTGCTAAGT
rGAPDH(812-1283)mRNA-R	GAPDH	Reverse	CGTTGCTGTCGAGACTTTATTG
rABCA3(1416-1755)mRNA-F	ABCA3	Forward	CAGAGCCATCATGCACTATCA
rABCA3(1416-1755)mRNA-R	ABCA3	Reverse	TTCACCTTGACGCAGAAGAG
rSFTPC(112-414)mRNA-F	SFTPC	Forward	TGCACCTCAAACGTCTTCTC
rSFTPC(112-414)mRNA-R	SFTPC	Reverse	GCTGTCTGGAGCCATCTTC
rSFTPB(1097-14651)mRNA-F	SFTPB	Forward	CATCCAAAGCCCTCACTTCT
rSFTPB(1097-1461)mRNA-R	SFTPB	Reverse	CCAGCCTCTCTCTTCTGTATTT
rHOPX(213-481)mRNA-F	HOPX	Forward	TGGAGATCCTGGAGTACAACT
rHOPX(213-481)mRNA-R	HOPX	Reverse	CACAGCATTACACTGCCAAAC
rAGER(1285-1533)mRNA-F	AGER	Forward	CCCTAGTACCTGAAGGACTCTT□
rAGER(1285-1533)mRNA-R	AGER	Reverse	GTGATGTTCTGACCACCTACTG
rAQP5(1208-1472)mRNA-F	AQP5	Forward	CACTGCTGGTACCACCTTATT
rAQP5(1208-1472)mRNA-R	AQP5	Reverse	CCTGCCTCATCTTCCTTTCTT
rMCIDAS(1713-1976)mRNA-F	MCIDAS	Forward	TGTGTGCTCTCAGCTACTTATG
rMCIDAS(1713-1976)mRNA-R	MCIDAS	Reverse	CAACTTCCTTAGGTTCCCTCTC
rTEKT2(378-578)mRNA-F	TEKT2	Forward	GGACAAGTGTCTGACGGATTTA
rTEKT2(378-578)mRNA-R	TEKT2	Reverse	CGATGACCTCCACCTCTTTATG
rSPDEF(1068-1416)mRNA-F	SPDEF	Forward	CACCTGGACATCTGGAAATCA
rSPDEF(1068-1416)mRNA-R	SPDEF	Reverse	CTTTCGGATGATGCCCTTCT
rMUC5AC(903-1216)mRNA-F	MUC5AC	Forward	CTCCAACACCATTCCTCCAA
rMUC5AC(903-1216)mRNA-R	MUC5AC	Reverse	GCCAAACACAGGCACAATC
rMUC5B(929-1179)mRNA-F	MUC5B	Forward	CCTAAGCTGTGCACCTACAA
rMUC5B(929-1179)mRNA-R	MUC5B	Reverse	TTTCGTTGGGCTGGATGAG
rSCGB1A1(121-324)mRNA-F	SCGB1A1	Forward	CGAGATTTGCACACGTCATTG
rSCGB1A1(121-324)mRNA-R	SCGB1A1	Reverse	CTACATACACAGTGGGCTCTTC
rSCGB3A2(160-414)mRNA-F	SCGB3A2	Forward	ACTCTGCTATTGCCTTCCTTATT
rSCGB3A2(160-414)mRNA-R	SCGB3A2	Reverse	CTGTCTTTCTCTTCCCTGATGTT
rGRIA1(3811-4084)mRNA-F	GRIA1	Forward	CACAGGTGGTACGGTGTATTT
rGRIA1(3811-4084)mRNA-R	GRIA1	Reverse	CACTGAGCTTGCATAGCATTTC
rGRHL2(2567-2871)mRNA-F	GRHL2	Forward	CGTCCCAAAGAGCCTGATAAA
rGRHL2(2567-2871)mRNA-R	GRHL2	Reverse	AGTAAACACGCCCACATACC
rSERPINE1(477-741)mRNA-F	SERPINE1	Forward	CCTGGAACAAGGATGAGATCAG
rSERPINE1(477-741)mRNA-R	SERPINE1	Reverse	CGTTGAAGTAGAGGGCATTCA
rESRP1(1540-1767)mRNA-F	ESRP1	Forward	GGCTGCACAGAAGTGTCATA
rESRP1(1540-1767)mRNA-R	ESRP1	Reverse	AAAGCACAGAGGGCTGATAAA
rCHGA(1686-1909)mRNA-F	CHGA	Forward	TCTCGACCCTGGAATATCCTT□
rCHGA(1686-1909)mRNA-R	CHGA	Reverse	CGAAGAGCCCAGAACAGATTTA
rCHGB(1769-2254)mRNA-F	CHGB	Forward	GGAAAGAGCAGGACAGAGATTAC
rCHGA(1769-2254)mRNA-R	CHGA	Reverse	GGAATCGTAGAAGTCGGGAAAC

REFERENCES

Al-Maary J, Eastwood MP, Russo FM, Deprest JA, Keijzer R. 2016. Fetal Tracheal Occlusion for Severe Pulmonary Hypoplasia in Isolated Congenital Diaphragmatic Hernia: A Systematic Review and Meta-analysis of Survival. *Annals of Surgery* **264**:929–933. doi:10.1097/SLA.0000000000001675

Aydin E, Joshi R, Oria M, Varisco BM, Lim FY, Peiro JL. 2018. Fetal tracheal occlusion in mice: a novel transuterine method. *The Journal of surgical research* **229**:311–315. doi:10.1016/j.jss.2018.04.028

Aydın E, Joshi R, Oria M, Lim F-Y, Varisco BM, Peiro JL. 2021a. Transuterine Fetal Tracheal Occlusion Model in Mice. *JoVE (Journal of Visualized Experiments)* e61772. doi:10.3791/61772

Aydın E, Torlak N, Yildirim A, Bozkurt EG. 2021b. Reversible Fetal Tracheal Occlusion in Mice: A Novel Minimal Invasive Technique. *Journal of Surgical Research* **260**:278–283. doi:10.1016/j.jss.2020.11.080

Boucherat O, Benachi A, Chailley-Heu B, Franco-Montoya ML, Elie C, Martinovic J, Bourbon JR. 2007. Surfactant maturation is not delayed in human fetuses with diaphragmatic hernia. *PLoS medicine* **4**:e237. doi:10.1371/journal.pmed.0040237

Chapin CJ, Ertsey R, Yoshizawa J, Hara A, Sbragia L, Greer JJ, Kitterman JA. 2005. Congenital diaphragmatic hernia, tracheal occlusion, thyroid transcription factor-1, and fetal pulmonary epithelial maturation. *American journal of physiology Lung cellular and molecular physiology* **289**:L44-52. doi:10.1152/ajplung.00342.2004

Chen J, Bardes EE, Aronow BJ, Jegga AG. 2009. ToppGene Suite for gene list enrichment analysis and candidate gene prioritization. *Nucleic Acids Research* **37**:W305-11. doi:10.1093/nar/gkp427

Coughlin MA, Werner NL, Gajarski R, Gadepalli S, Hirschl R, Barks J, Treadwell MC, Ladino-Torres M, Kreutzman J, Mychaliska GB. 2015. Prenatally diagnosed severe CDH: mortality and morbidity remain high. *Journal of pediatric surgery*. doi:10.1016/j.jpedsurg.2015.10.082

Cruz-Martinez R, Moreno-Alvarez O, Prat J, Krauel L, Tarrado X, Castanon M, Hernandez-Andrade E, Albert A, Gratacos E. 2009. Lung tissue blood perfusion changes induced by in utero tracheal occlusion in a rabbit model of congenital diaphragmatic hernia. *Fetal diagnosis and therapy* **26**:137–42. doi:10.1159/000254485

Davey MG, Biard JM, Robinson L, Tsai J, Schwarz U, Danzer E, Adzick NS, Flake AW, Hedrick HL. 2005. Surfactant protein expression is increased in the ipsilateral but not contralateral lungs of fetal sheep with left-sided diaphragmatic hernia. *Pediatric pulmonology* **39**:359–67. doi:10.1002/ppul.20175

Dobin A, Davis CA, Schlesinger F, Drenkow J, Zaleski C, Jha S, Batut P, Chaisson M, Gingeras TR. 2013. STAR: ultrafast universal RNA-seq aligner. *Bioinformatics* **29**:15–21. doi:10.1093/bioinformatics/bts635

Dobrinskikh E, Al-Juboori SI, Oria M, Reisz JA, Zheng C, Peiro JL, Marwan AI. 2020. Heterogeneous Response in Rabbit Fetal Diaphragmatic Hernia Lungs After Tracheal Occlusion. *J Surg Res* **250**:23–38. doi:10.1016/j.jss.2019.12.025

Du Y, Guo M, Whitsett JA, Xu Y. 2015. "LungGENS": a web-based tool for mapping single-cell gene expression in the developing lung. *Thorax* ePub ahead of print. doi:10.1136/thoraxjnl-2015-207035

Endale M, Ahlfeld S, Bao E, Chen X, Green J, Bess Z, Weirauch MT, Xu Y, Perl AK. 2017. Temporal, spatial, and phenotypical changes of PDGFRalpha expressing fibroblasts during late lung development. *Developmental biology*. doi:10.1016/j.ydbio.2017.03.020

Engels AC, Brady PD, Kammoun M, Finalet Ferreiro J, DeKoninck P, Endo M, Toelen J, Vermeesch JR, Deprest J. 2016. Pulmonary transcriptome analysis in the surgically induced rabbit model of diaphragmatic hernia treated with fetal tracheal occlusion. *Disease models & mechanisms* **9**:221–8. doi:10.1242/dmm.021626

Harrison MR, Jester JA, Ross NA. 1980. Correction of congenital diaphragmatic hernia in utero. I. The model: intrathoracic balloon produces fatal pulmonary hypoplasia. *Surgery* **88**:174–82.

Joshi R, Liu S, Brown MD, Young SM, Batie M, Kofron JM, Xu Y, Weaver TE, Apsley K, Varisco BM. 2016. Stretch regulates expression and binding of chymotrypsin-like elastase 1 in the postnatal lung. *FASEB journal : official publication of the Federation of American Societies for Experimental Biology* **30**:590–600. doi:10.1096/fj.15-277350

Kassambara A. 2020. ggpubr: Publication Ready Plots - Articles - STHDA. http://www.sthda.com/english/articles/24-ggpubr-publication-ready-plots/

Kassambara A. 2020. rstatix: Pipe-Friendly Framework for Basic Statistical Tests.

Krämer A, Green J, Pollard J, Tugendreich S. 2014. Causal analysis approaches in Ingenuity Pathway Analysis. *Bioinformatics* **30**:523–530. doi:10.1093/bioinformatics/btt703

Lange AW, Sridharan A, Xu Y, Stripp BR, Perl AK, Whitsett JA. 2015. Hippo/Yap signaling controls epithelial progenitor cell proliferation and differentiation in the embryonic and adult lung. *J Mol Cell Biol* **7**:35–47. doi:10.1093/jmcb/mju046

Love MI, Huber W, Anders S. 2014. Moderated estimation of fold change and dispersion for RNA-seq data with DESeq2. *Genome Biology* **15**:550. doi:10.1186/s13059-014-0550-8

Mahoney JE, Mori M, Szymaniak AD, Varelas X, Cardoso WV. 2014. The hippo pathway effector Yap controls patterning and differentiation of airway epithelial progenitors. *Developmental cell* **30**:137–50. doi:10.1016/j.devcel.2014.06.003

Meng Z, Moroishi T, Guan KL. 2016. Mechanisms of Hippo pathway regulation. *Genes & development* **30**:1–17. doi:10.1101/gad.274027.115

Nantie LB, Young RE, Paltzer WG, Zhang Y, Johnson RL, Verheyden JM, Sun X. 2018. Lats inactivation reveals hippo function in alveolar type I cell differentiation during lung transition to air breathing. *Development (Cambridge, England)*. doi:10.1242/dev.163105

Nguyen TM, van der Merwe J, Elowsson Rendin L, Larsson-Callerfelt A-K, Deprest J, Westergren-Thorsson G, Toelen J. 2021. Stretch increases alveolar type 1 cell number in fetal lungs through ROCK-Yap/Taz pathway. *Am J Physiol Lung Cell Mol Physiol* **321**:L814–L826. doi:10.1152/ajplung.00484.2020

Peiro JL, Oria M, Aydin E, Joshi R, Cabanas N, Schmidt R, Schroeder C, Marotta M, Varisco BM. 2018. Proteomic profiling of tracheal fluid in an ovine model of congenital diaphragmatic hernia and fetal tracheal occlusion. *Am J Physiol Lung Cell Mol Physiol*

315:L1028–L1041. doi:10.1152/ajplung.00148.2018
Penkala IJ, Liberti DC, Pankin J, Sivakumar A, Kremp MM, Jayachandran S, Katzen J, Leach JP, Windmueller R, Stolz K, Morley MP, Babu A, Zhou S, Frank DB, Morrisey EE. 2021. Age-dependent alveolar epithelial plasticity orchestrates lung homeostasis and regeneration. *Cell Stem Cell*. doi:10.1016/j.stem.2021.04.026
Prat Ortells J, Albert A, Tarrado X, Krauel L, Cruz R, Moreno-Alvarez O, Fuste V, Castanon M. 2014. Airway and vascular maturation stimulated by tracheal occlusion do not correlate in the rabbit model of diaphragmatic hernia. *Pediatric research* **75**:487–92. doi:10.1038/pr.2013.244
R Core Team. 2019. R: A Language and Environment for Statistical Computing. Vienna, Austria: R Foundation for Statistical Computing.
Riccetti M, Gokey JJ, Aronow B, Perl A-KT. 2020. The elephant in the lung: Integrating lineage-tracing, molecular markers, and single cell sequencing data to identify distinct fibroblast populations during lung development and regeneration. *Matrix Biol*. doi:10.1016/j.matbio.2020.05.002
Soldt BJ van, Qian J, Li J, Tang N, Lu J, Cardoso WV. 2019. Yap and its subcellular localization have distinct compartment-specific roles in the developing lung. *Development* **146**. doi:10.1242/dev.175810
Tan SY, Krasnow MA. 2016. Developmental origin of lung macrophage diversity. *Development (Cambridge, England)* **143**:1318–27. doi:10.1242/dev.129122
Varisco BM, Sbragia L, Chen J, Scorletti F, Joshi R, Wong HR, Lopes-Figueira R, Oria M, Peiro J. 2016. Excessive Reversal of Epidermal Growth Factor Receptor and Ephrin Signaling Following Tracheal Occlusion in Rabbit Model of Congenital Diaphragmatic Hernia. *Mol Med* **22**. doi:10.2119/molmed.2016.00121
Vaughan AE, Brumwell AN, Xi Y, Gotts JE, Brownfield DG, Treutlein B, Tan K, Tan V, Liu FC, Looney MR, Matthay MA, Rock JR, Chapman HA. 2015. Lineage-negative progenitors mobilize to regenerate lung epithelium after major injury. *Nature* **517**:621–5. doi:10.1038/nature14112
Vuckovic A, Herber-Jonat S, Flemmer AW, Ruehl IM, Votino C, Segers V, Benachi A, Martinovic J, Nowakowska D, Dzieniecka M, Jani JC. 2016. Increased TGF-beta: a drawback of tracheal occlusion in human and experimental congenital diaphragmatic hernia? *American journal of physiology Lung cellular and molecular physiology* **310**:L311-27. doi:10.1152/ajplung.00122.2015
Yang Y, Riccio P, Schotsaert M, Mori M, Lu J, Lee DK, Garcia-Sastre A, Xu J, Cardoso WV. 2018. Spatial-Temporal Lineage Restrictions of Embryonic p63(+) Progenitors Establish Distinct Stem Cell Pools in Adult Airways. *Developmental cell* **44**:752-761.e4. doi:10.1016/j.devcel.2018.03.001
Zuo W, Rostami MR, Shenoy SA, LeBlanc MG, Salit J, Strulovici-Barel Y, O'Beirne SL, Kaner RJ, Leopold PL, Mezey JG, Schymeinsky J, Quast K, Visvanathan S, Fine JS, Thomas MJ, Crystal RG. 2020. Cell-specific expression of lung disease risk-related genes in the human small airway epithelium. *Respiratory Research* **21**:200. doi:10.1186/s12931-020-01442-9
